# Supplementary material for: The Cancer-Associated Genetic Variant Rs3903072 Modulates Immune Cells in the Tumor Microenvironment
Source: Front Genet. 2019 Aug 23;10:754. doi: 10.3389/fgene.2019.00754 (PMC6715770; doi:10.3389/fgene.2019.00754)
Supplement: Supplementary file 1 [file DataSheet_1.pdf]

Supplementary Information for

The cancer-associated genetic variant rs3903072 modulates immune cells in the tumor microenvironment

Yi Zhang[1,2], Mohith Manjunath[2], Jialu Yan[2,3], Brittany A. Baur[4], Shilu Zhang[4], Sushmita Roy[4,5], and Jun S. Song[2,3,*]

[1]Department of Bioengineering, University of Illinois at Urbana-Champaign, Urbana, IL 61801, USA
[2]Carl R. Woese Institute for Genomic Biology, University of Illinois at Urbana-Champaign, Urbana, IL 61801, USA
[3]Department of Physics, University of Illinois at Urbana-Champaign, Urbana, IL 61801, USA
[4]Wisconsin Institute for Discovery, University of Wisconsin–Madison, Madison, WI 53792, USA
[5]Department of Biostatistics and Medical Informatics, University of Wisconsin–Madison, Madison, WI 53792, USA

*** Correspondence:**
Jun S. Song (songj@illinois.edu)

1 Supplementary Methods

Genome-wide association studies (GWAS) variants

Using data from *Michailidou et al.* (Michailidou et al., 2013) and the NHGRI-EBI GWAS catalog (MacArthur et al., 2017), we first obtained variants associated with estrogen receptor-positive (ER+) breast cancer. To identify the variants associated with immuno-inflammatory traits, we used the NHGRI-EBI GWAS catalog v1.0.2 and the annotation table from the GWAS catalog mapping the GWAS traits to the parent disease category using information from the Experimental Factor Ontology (EFO) database (Malone et al., 2010). Using the "Parent term" column, we first selected SNP-trait associations from the GWAS catalog containing the term "Immune system disorder" or "Inflammatory measurement". We also included additional traits in other disease categories with recursive ontology parents containing the keyword "immun" or

“inflamm”. For example, Crohn’ disease was categorized as a digestive system disorder in the GWAS EFO mapping file, but we included it in our study since it is under the “inflammatory bowel disease” category in the EFO ontology database. Finally, we obtained a set of 3404 SNPs associated with immuno-inflammatory traits. For each ER+ breast cancer GWAS SNP, we scanned the proximal region using a moving window of length 100 kb containing the SNP and counted the number of immuno-inflammatory GWAS SNPs within each window. The maximum number of immuno-inflammatory SNPs in these running windows was then recorded for each breast cancer GWAS SNP. When the breast cancer GWAS SNPs were ranked based on these maximum counts, rs3903072 emerged as the top SNP.

A supplementary table from *Michailidou et al.* (Michailidou et al., 2017) provides a list of SNPs associated with breast cancer with p-value less than 10^{-5}. However, as *Michailidou et al.* (Michailidou et al., 2017) used the p-value threshold of 10^{-8} in the final report, we mainly focused on rs3903072 ($p = 2 \times 10^{-12}$) rather than the nearby breast cancer GWAS SNP rs617791 showing a weaker association ($p = 7 \times 10^{-6}$, **Figure 1A**).

Genotype imputation for TCGA datasets

For genotype imputation of Breast Invasive Carcinoma (BRCA), Uterine Corpus Endometrial Carcinoma (UCEC), Head-Neck Squamous Cell Carcinoma (HNSC) and Low Grade Glioma (LGG) datasets from TCGA, raw genotypes with genotype confidence score greater than 0.1 in birdseed format were marked as missing genotypes to be imputed along with the non-probed SNPs. The filtered genotypes were then imputed using the Michigan Imputation Server (Das et al., 2016), selecting the Haplotype Reference Consortium (HRC) r1.1 2016 (Loh et al., 2016a) as

a reference panel, Eagle v2.3 (Loh et al., 2016b) for phasing, and EUR population as the quality control option.

The Genotype-Tissue Expression (GTEx) project data

The GTEx (GTEx Consortium, 2013; Carithers et al., 2015) gene expression levels in RPKM (Reads Per Kilobase Million) were downloaded from the GTEx portal (file: GTEx_Analysis_v6_RNA-seq_RNA-SeQCv1.1.8_gene_rpkm.gct), and the annotations for the samples and tissues were obtained from GTEx_Data_V6_Annotations_SampleAttributesDS.txt. The mean *CTSW* expression levels were computed for **Figure 2D**; the tissue-wise distributions of gene expression levels of all the genes near *CTSW* were shown using the UCSC GTEx track (Kent et al., 2002) (**Supplementary Figure 5**).

Expression quantitative trait loci (eQTL) analysis for TCGA-BRCA

Among ER+ breast cancer patients, we performed eQTL analysis by constructing a multivariate linear model, with the genotypes at the GWAS SNP rs3903072 and the gene copy number (CN) (Zhang et al., 2018b) as the predictors and the gene expression levels as the response variable. The gene expression levels in FPKM were log-transformed as $log_2(FPKM + 1)$ and taken to be the response. The SNP genotypes were encoded as the number of risk alleles (0, 1 or 2), and the gene CN was computed by taking gene length-weighted average of tumor CN segmentation data, transforming the segmentation unit back to the CN unit ($CN = 2 \times 2^{segmentation}$). Multivariate linear regression was then performed for each gene within the 3 Mb region centered at rs3903072. Genes with $\overline{FPKM} \geq 1$ ($\overline{FPKM}$: mean expression among tumor samples) and the genotype p-value ≤ 0.05 from the linear regression were selected for further investigation.

Among them, *CTSW* was found to have a different trend compared to other genes, as described in the main manuscript; the three significant eQTL genes near *CTSW – FIBP, MUS81 and EIF1AD* – were then selected for comparison with *CTSW* in **Figure 1B**, because of their strong eQTL correlation that was also observed in an earlier study (Michailidou et al., 2013).

In other cancer types and normal tissues, eQTL analyses were performed using BRCA, UCEC, HNSC, LGG and GTEx data, taking the genotype status at rs3903072 to be the predictor and *CTSW* expression as the response. All RNA-seq FPKM values from TCGA were log-transformed as in BRCA. For GTEx, the normalized gene expression matrices of whole blood and breast mammary tissues were downloaded from GTEx Analysis V7 release (dbGaP Accession phs000424.v7.p2).

TCGA survival analysis

Survival analysis in TCGA ER+ breast cancer patients was performed using the clinical data obtained from TCGA Genomic Data Commons (GDC). The differences in survival rate between the two breast cancer patient groups separated by *CTSW* median expression level was tested using log-rank test with the R packages survival (Therneau, 2015) and survminer (Kassambara and Kosinski, 2017). Survival analysis results were also obtained in endometrial cancer (UCEC), head and neck cancer (HNSC), and renal cancer were obtained from the human protein atlas (THPA) (Uhlen et al., 2017), where the median expression level of *CTSW* was chosen as the cutoff threshold for grouping patients. The relevant data was obtained from the following webpages for UCEC, HNSC and renal cancer (UCEC: https://www.proteinatlas.org/ENSG00000172543-CTSW/pathology/tissue/endometrial+cancer;

HNSC: https://www.proteinatlas.org/ENSG00000172543-CTSW/pathology/tissue/head+and+neck+cancer; Renal cancer: https://www.proteinatlas.org/ENSG00000172543-CTSW/pathology/tissue/renal+cancer). The three datasets in renal cancer were also checked separately, including Kidney Renal Clear Cell Carcinoma (KIRC; https://www.proteinatlas.org/ENSG00000172543-CTSW/pathology/tissue/renal+cancer/KIRC), Kidney Renal Papillary Cell Carcinoma (KIRP; https://www.proteinatlas.org/ENSG00000172543-CTSW/pathology/tissue/renal+cancer/KIRP), and Kidney Chromophobe (KICH; https://www.proteinatlas.org/ENSG00000172543-CTSW/pathology/tissue/renal+cancer/KICH).

CTSW expression and promoter transcription activity

We used BioGPS GeneAtlas (Wu et al., 2016), cancer cell line encyclopedia (CCLE) (Barretina et al., 2012) and functional annotation of the mammalian genome (FANTOM) (The Fantom Consortium and the Riken PMI and CLST (DGT) et al., 2014) resources for analyzing the gene expression levels of *CTSW* across tissues and cell lines. Microarray gene expression data for *CTSW* were directly downloaded from the BioGPS web resource. There were 176 samples including replicates, and the mean expression of replicates was calculated for each tissue. CCLE gene expression data in RPKM units from RNA-seq data of 1156 samples were downloaded from the CCLE website (file: CCLE_DepMap_18q3_RNAseq_RPKM_20180718.gct). FANTOM gene expression data in TPM units was obtained from the FANTOM web resource (file: hg19.gene_phase1and2combined_tpm.osc.txt). The FANTOM data consisted of 1829 samples (tissue and cell type information obtained from HumanSamples2.0.sdrf.xlsx).

Chromatin accessibility at CTSW promoter

Encyclopedia of DNA Elements (ENCODE) (The Encode Project Consortium et al., 2012) and the Roadmap Epigenomics project (Bernstein et al., 2010) consortiums have generated various tissue-specific DNase I hypersensitive sites sequencing (DNase-seq) chromatin accessibility data. For the ENCODE data, the DNase I hypersensitivity sites (DHS) tracks of all cell types in ENCODE Tier 1 were displayed, together with the three cell lines related to breast tissue (HMEC, MCF-7, T-47D). We also examined all cell types in ENCODE Tier 2 and Tier 3, selecting the ones with a DHS at *CTSW* promoter to include in **Figure 2C**. For the Roadmap Epigenomics data, we displayed the wiggle track of the first DNase-seq replicate in each cell type (**Figure 2C**).

Random forest regression approach for predicting high-resolution chromatin contact counts

To predict high-resolution Hi-C interactions around the GWAS SNP rs3903072 in Natural Killer cells, T cells and vHMEC, we trained a local random forest regression model within1 Mb of the SNP, using an approach similar to our previously published method (Zhang et al., 2018a). vHMEC serves as a control cell line, since it is a homogeneous, non-cancerous mammary epithelial cell line and does not contain other cell types such as T cells and NK cells. We trained the models on region-pairs involving the chr11:65580000-65585000 (hg19) 5 kb bin, which overlaps the GWAS SNP and PRE1, and 5 kb bins within 1 Mb from the SNP, using published high-resolution (5 kb) Hi-C datasets in five different cell lines (Rao et al., 2014) and complementary one-dimensional signals as features; these signals were histone marks, DNase I and DNase I accessible sequence specific motifs for CTCF, RAD21 and TBP (Sherwood et al., 2014). Histone marks and DNase I data were obtained from ENCODE and the Roadmap Epigenomics Project for the five training (GM12878, K562, HUVEC, NHEK, HMEC) and three

test (NK, T cells and vHMEC) cell lines. Since histone datasets for all the features were not available in vHMEC, we used imputed signals from the Avocado pipeline (Schreiber et al., 2018). Data processing and normalization were done as described in Zhang *et al.* (Zhang et al., 2018a) and included normalization for sequencing depth and collapsing replicates by median. To account for overall differences in signal across cell lines, we additionally discretized each of 5 kb ChIP-seq signals using k-means clustering with k=20.

A region was represented as a 10-dimensional feature vector, each dimension corresponding to one of the 10 genome-wide datasets (6 histone ChIP-seq, DNase-seq and 3 DNase-seq derived motifs). Features for a pair of regions were obtained by concatenating the 10-dimensional feature vectors of the two regions together with the feature vector of the intervening region between the two regions and the distance between the two regions to obtain a feature vector of size 31. The feature associated with the intervening region was the mean signal value of the features in the region for ChIP-seq and DHS, similar to the “WINDOW” feature in TargetFinder (Whalen et al., 2016). Once trained, we used the models to generate contact count predictions in the 1 Mb region in NK cells, CD8+ αβ T cells and vHMEC, using feature datasets from the Roadmap Epigenomics database.

GREAT analysis

ChIP-seq data for the transcription factor TEAD2 were obtained from ENCODE (accession numbers: ENCFF777YSR and ENCFF828PUB). A *q*-value threshold of 0.05 was chosen to obtain a set of high-confidence peaks. The resulting peak regions were then used as input to GREAT with default settings (McLean et al., 2010).

2 Supplementary Tables

Supplementary Table 1. GWAS traits around rs3903072 for the region shown in **Figure 1A**.

GWAS SNP ID	Trait Category	GWAS traits reported in each study	PUBMED ID for each study
rs10750766	Blood cells-related	Diastolic blood pressure x alcohol consumption interaction (2df test) ; High light scatter reticulocyte count ; High light scatter reticulocyte percentage of red cells ; Immature fraction of reticulocytes ; Systolic blood pressure x alcohol consumption interaction (2df test)	29912962 ; 27863252 ; 27863252 ; 27863252 ; 29912962
rs10791824	Immuno-inflammatory	Atopic dermatitis	26482879
rs10896045	Blood cells-related	Blood protein levels	29875488
rs11227302	Immuno-inflammatory	Systemic lupus erythematosus	28714469
rs11227306	Other traits	DNA methylation (variation)	23725790
rs11602769	Other traits	Allergic sensitization	30013184
rs11604462	Other traits	Glomerular filtration rate (creatinine)	28452372
rs118086960	Immuno-inflammatory	Psoriasis	28537254
rs12223803	Blood cells-related	Albumin-globulin ratio	29403010
rs12576766	Other traits	Serum uric acid levels	29403010
rs185542523	Other traits	Maximum cranial width	29698431
rs201316070	Other traits	Systolic blood pressure x smoking status (current vs non-current) interaction (2df test) ; Systolic blood pressure x smoking status (ever vs never) interaction (2df test)	29455858 ; 29455858
rs2231884	Immuno-inflammatory	Inflammatory bowel disease	23128233
rs3825068	Blood cells-related	Blood protein levels	29875488
rs3903072	Breast cancer	Breast cancer ; Breast cancer ; Breast cancer	23535729 ; 25751625 ; 29059683
rs4014195	Other traits	Chronic kidney disease ; Glomerular filtration rate (creatinine)	20383146 ; 26831199
rs478304	Immuno-inflammatory	Acne (severe) ; Spherical equivalent or myopia (age of diagnosis)	24927181 ; 29808027

rs479844	Other traits	Allergic disease (asthma, hay fever or eczema) ; Atopic dermatitis ; Atopic dermatitis ; Atopic march	29083406 ; 22197932 ; 26482879 ; 26542096
rs489574	Immuno-inflammatory	Systemic lupus erythematosus	28714469
rs494003	Immuno-inflammatory	Systemic lupus erythematosus ; Systemic lupus erythematosus	26502338 ; 27399966
rs526631	Blood cells-related	Eosinophil percentage of granulocytes ; Neutrophil percentage of granulocytes	27863252 ; 27863252
rs568617	Immuno-inflammatory	Chronic inflammatory diseases (ankylosing spondylitis, Crohn's disease, psoriasis, primary sclerosing cholangitis, ulcerative colitis) (pleiotropy) ; Crohn's disease	26974007 ; 26192919
rs5792377	Other traits	Heel bone mineral density	30048462
rs593982	Immuno-inflammatory	Atopic dermatitis	23042114
rs617791	Other traits	Breast cancer	29059683
rs634534	Blood cells-related	Eosinophil counts ; Sum eosinophil basophil counts	27863252 ; 27863252
rs637571	Blood cells-related	Eosinophil percentage of white cells	27863252
rs642803	Other traits	Educational attainment (MTAG) ; Highest math class taken (MTAG) ; Urate levels	30038396 ; 30038396 ; 23263486
rs7123489	Other traits	Creatinine levels	29403010
rs72941051	Other traits	Diastolic blood pressure x smoking status (current vs non-current) interaction (2df test) ; Diastolic blood pressure x smoking status (ever vs never) interaction (2df test) ; Systolic blood pressure x smoking status (current vs non-current) interaction (2df test) ; Systolic blood pressure x smoking status (ever vs never) interaction (2df test)	29455858 ; 29455858 ; 29455858 ; 29455858
rs77291001	Other traits	Maximum cranial width	29698431
rs77779142	Immuno-inflammatory	Rosacea symptom severity*	29771307
rs9795139	Other traits	Serum uric acid levels	29403010

* rs77779142 is not shown in **Figure 1A**, because Rosacea symptom was not listed as an immune-related disease in the original GWAS annotation; it is recorded here as an immuno-inflammatory variant, since Rosacea is an inflammatory skin condition.

Supplementary Table 2. Genetic linkage between rs3903072 and the nearby immuno-inflammatory variants.

Immuno-inflammatory SNP	r^2	D'	Immuno-inflammatory risk allele	Immuno-inflammatory risk allele frequency	Allele correlated with rs3903072-G (risk)
rs478304	0.0598	0.2474	T	0.534	Linkage Equilibrium
rs593982	0.0227	0.4476	C	0.883	Linkage Equilibrium
rs494003	0.1078	0.737	A	0.189	A
rs489574	0.0003	0.0226	A	0.343	Linkage Equilibrium
rs479844	0.3262	0.6931	G	0.557	A
rs11227302	0.1057	0.7416	A	0.184	A
rs10791824	0.3234	0.7157	G	0.575	A
rs118086960	0.0233	0.1553	T	0.532	Linkage Equilibrium
rs77779142	0.1673	1.0000	T	0.164	T
rs568617	0.1851	0.9657	T	0.189	T
rs2231884	0.1586	0.9737	T	0.164	T

Supplementary Table 3. List of eQTL genes correlated with the rs3903072 genotype within 3 Mb of the SNP.

Available as a separate supplementary file.

Supplementary Table 4. Annotation of genes in the rs3903072-*CTSW* region from PANTHER(Mi et al., 2017).

**Gene ID**	**Mapped IDs**	**Gene Name; Gene Symbol**	**PANTHER Family/Subfamily**	**PANTHER Protein Class**
HUMAN|HGNC=25104|UniProtKB=Q2VPB7	AP5B1	AP-5 complex subunit beta-1;AP5B1	AP-5 COMPLEX SUBUNIT BETA-1 (PTHR34033:SF1)	
HUMAN|HGNC=24144|UniProtKB=Q96A11	GAL3ST3	Galactose-3-O-sulfotransferase 3;GAL3ST3	GALACTOSE-3-O-SULFOTRANSFERASE 3 (PTHR14647:SF76)	
HUMAN|HGNC=13718|UniProtKB=P15407	FOSL1	Fos-related antigen 1;FOSL1	FOS-RELATED ANTIGEN 1 (PTHR23351:SF6)	basic leucine zipper transcription factor(PC00056)
HUMAN|HGNC=5275|UniProtKB=Q92993	KAT5	Histone acetyltransferase KAT5;KAT5	HISTONE ACETYLTRANSFERASE KAT5 (PTHR10615:SF124)	acetyltransferase(PC00038);chromatin/chromatin-binding protein(PC00077);zinc finger transcription factor(PC00244)
HUMAN|HGNC=26423|UniProtKB=Q86XE0	SNX32	Sorting nexin-32;SNX32	SORTING NEXIN-32 (PTHR45850:SF3)	
HUMAN|HGNC=3705|UniProtKB=O43427	FIBP	Acidic fibroblast growth factor intracellular-binding protein;FIBP	ACIDIC FIBROBLAST GROWTH FACTOR INTRACELLULAR-BINDING PROTEIN (PTHR13223:SF2)	
HUMAN|HGNC=26555|UniProtKB=Q3SY00	TSGA10IP	Testis-specific protein 10-interacting protein;TSGA10IP	TESTIS-SPECIFIC PROTEIN 10-INTERACTING PROTEIN (PTHR21501:SF5)	
HUMAN|HGNC=10769|UniProtKB=Q13435	SF3B2	Splicing factor 3B subunit 2;SF3B2	SPLICING FACTOR 3B SUBUNIT 2 (PTHR12785:SF6)	
HUMAN|HGNC=2478|UniProtKB=Q15828	CST6	Cystatin-M;CST6	CYSTATIN-M (PTHR47033:SF1)	

HUMAN|HGNC=2546|UniProtKB=P56202	CTSW	Cathepsin W;CTSW	CATHEPSIN W (PTHR12411:SF101)	cysteine protease(PC00081);protease inhibitor(PC00191)
HUMAN|HGNC=10538|UniProtKB=O43290	SART1	U4/U6.U5 tri-snRNP-associated protein 1;SART1	U4/U6.U5 TRI-SNRNP-ASSOCIATED PROTEIN 1 (PTHR14152:SF5)	extracellular matrix protein(PC00102)
HUMAN|HGNC=8525|UniProtKB=O14753	OVOL1	Putative transcription factor Ovo-like 1;OVOL1	TRANSCRIPTION FACTOR OVO-LIKE 1-RELATED (PTHR10032:SF217)	
HUMAN|HGNC=17116|UniProtKB=Q8NEC5	CATSPER1	Cation channel sperm-associated protein 1;CATSPER1	CATION CHANNEL SPERM-ASSOCIATED PROTEIN 1 (PTHR47193:SF1)	
HUMAN|HGNC=24116|UniProtKB=Q8TDP1	RNASEH2C	Ribonuclease H2 subunit C;RNASEH2C	RIBONUCLEASE H2 SUBUNIT C (PTHR47063:SF1)	
HUMAN|HGNC=1874|UniProtKB=P23528	CFL1	Cofilin-1;CFL1	COFILIN-1 (PTHR11913:SF17)	non-motor actin binding protein(PC00165)
HUMAN|HGNC=2482|UniProtKB=P04080	CST6	Cystatin-B;CSTB	CYSTATIN-B (PTHR11414:SF22)	cysteine protease inhibitor(PC00082)
HUMAN|HGNC=17397|UniProtKB=O75531	BANF1	Barrier-to-autointegration factor;BANF1	BARRIER-TO-AUTOINTEGRATION FACTOR (PTHR12912:SF10)	
HUMAN|HGNC=30032|UniProtKB=Q6VY07	PACS1	Phosphofurin acidic cluster sorting protein 1;PACS1	PHOSPHOFURIN ACIDIC CLUSTER SORTING PROTEIN 1 (PTHR13280:SF16)	
HUMAN|HGNC=28147|UniProtKB=Q8N9N8	EIF1AD	Probable RNA-binding protein EIF1AD;EIF1AD	RNA-BINDING PROTEIN EIF1AD-RELATED (PTHR21641:SF0)	
HUMAN|HGNC=3219|UniProtKB=O95967	EFEMP2	EGF-containing fibulin-like extracellular	EGF-CONTAINING FIBULIN-LIKE EXTRACELLULAR	annexin(PC00050);calmodulin(PC00061);cell adhesion

		matrix protein 2;EFEMP2	MATRIX PROTEIN 2 (PTHR24034:SF96)	molecule(PC00069);extracellular matrix glycoprotein(PC00100);extracellular matrix structural protein(PC00103);signaling molecule(PC00207)
HUMAN|HGNC=28801|UniProtKB=Q9H3H3	C11orf68	UPF0696 protein C11orf68;C11orf68	UPF0696 PROTEIN C11ORF68 (PTHR31977:SF1)	
HUMAN|HGNC=29814|UniProtKB=Q96NY9	MUS81	Crossover junction endonuclease MUS81;MUS81	CROSSOVER JUNCTION ENDONUCLEASE MUS81 (PTHR13451:SF0)	

Supplementary Table 5. List of DNase-seq peak files for lymphocytes.

**Cell line (treatment)**	**ENCODE accessions numbers of DHS peak files**
Cd4+, helper T cell	ENCFF569GSL, ENCFF907KBL, ENCFF988HSM
Cd8+, alpha-beta T cell	ENCFF071RMN, ENCFF422HNI, ENCFF662NTN
Jurkat clone E61	ENCFF582KJR, ENCFF837AOM
Natural killer cell	ENCFF224LJW, ENCFF933OXV
T cell	ENCFF026SFK, ENCFF286UIJ, ENCFF304TBE, ENCFF345YDG, ENCFF923EVD
B cell	ENCFF210RAG, ENCFF654IWG, ENCFF772OPR
T helper17 cell	ENCFF001WCL, ENCFF001WTC
T helper1 cell	ENCFF434LIX, ENCFF001WCS, ENCFF001WTE, ENCFF001WTI, ENCFF001WTL, ENCFF773IYG, ENCFF001WCQ, ENCFF001WTF, ENCFF001WTM
T helper2 cell	ENCFF570MGY, ENCFF001WCW, ENCFF001WTO, ENCFF001WTS, ENCFF001WTU, ENCFF001WCU, ENCFF001WTQ
Common myeloid progenitor, CD34+	ENCFF037XOG, ENCFF387SIU, ENCFF401NSY, ENCFF457SNT, ENCFF479XZN, ENCFF499OEI, ENCFF600EJV, ENCFF664WUJ, ENCFF686ZXP, ENCFF727NEX, ENCFF770BVB, ENCFF918ICP, ENCFF182JTX, ENCFF264UIE, ENCFF927MCZ

Supplementary Table 6. GREAT analysis results showing various ontology associated terms for cis-regulatory activities of TEAD2.

Available as a separate supplementary file.

3 Supplementary Figures

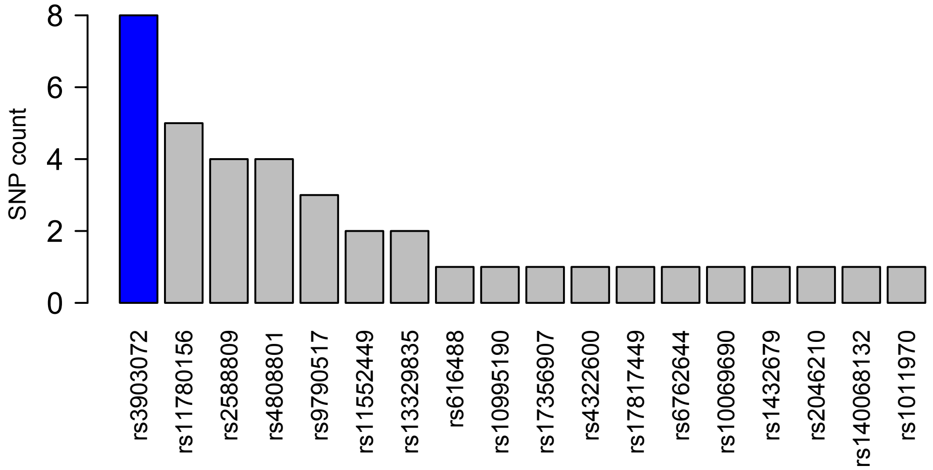

Supplementary Figure 1. Number of GWAS variants associated with immuno-inflammatory traits around each ER+ breast cancer GWAS SNP. Only those breast cancer GWAS SNPs with a non-zero count of immuno-inflammatory SNPs within +/- 100 kb are shown.

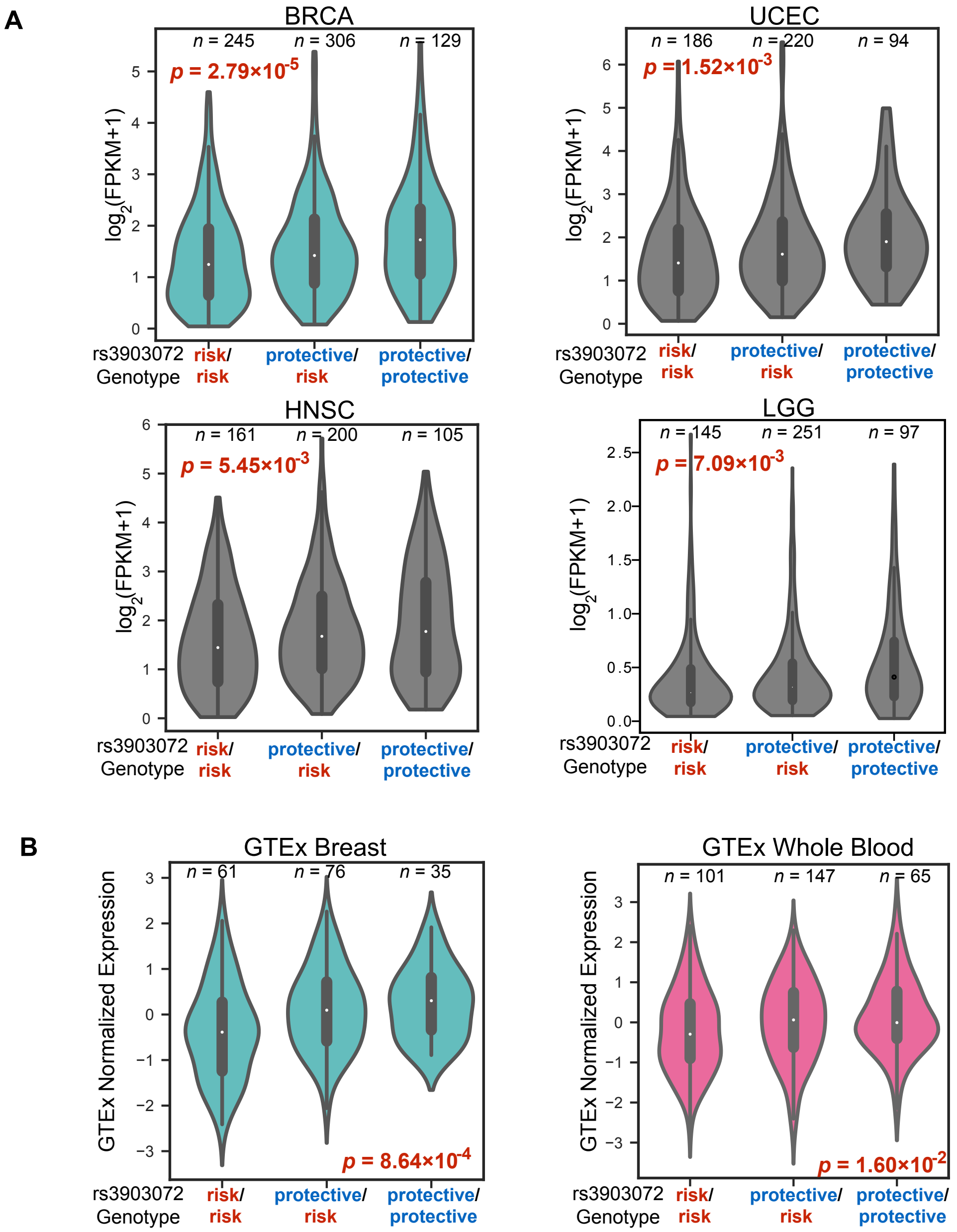

Supplementary Figure 2. Violin plots for the eQTL analysis in different datasets. A linear model was constructed between the *CTSW* expression level and the genotype status at the GWAS SNP rs3903072; the *p*-values shown are for the linear coefficient of genotype. Gene copy number is not included in the model for this figure. (**A**) eQTL analysis in cancers from TCGA, using ER+ breast cancer subtype in BRCA, endometrial cancer (UCEC), head and neck cancer (HNSC), and low grade glioma (LGG). (**B**) eQTL analysis in normal tissues from GTEx, using mammary tissue and whole blood tissue.

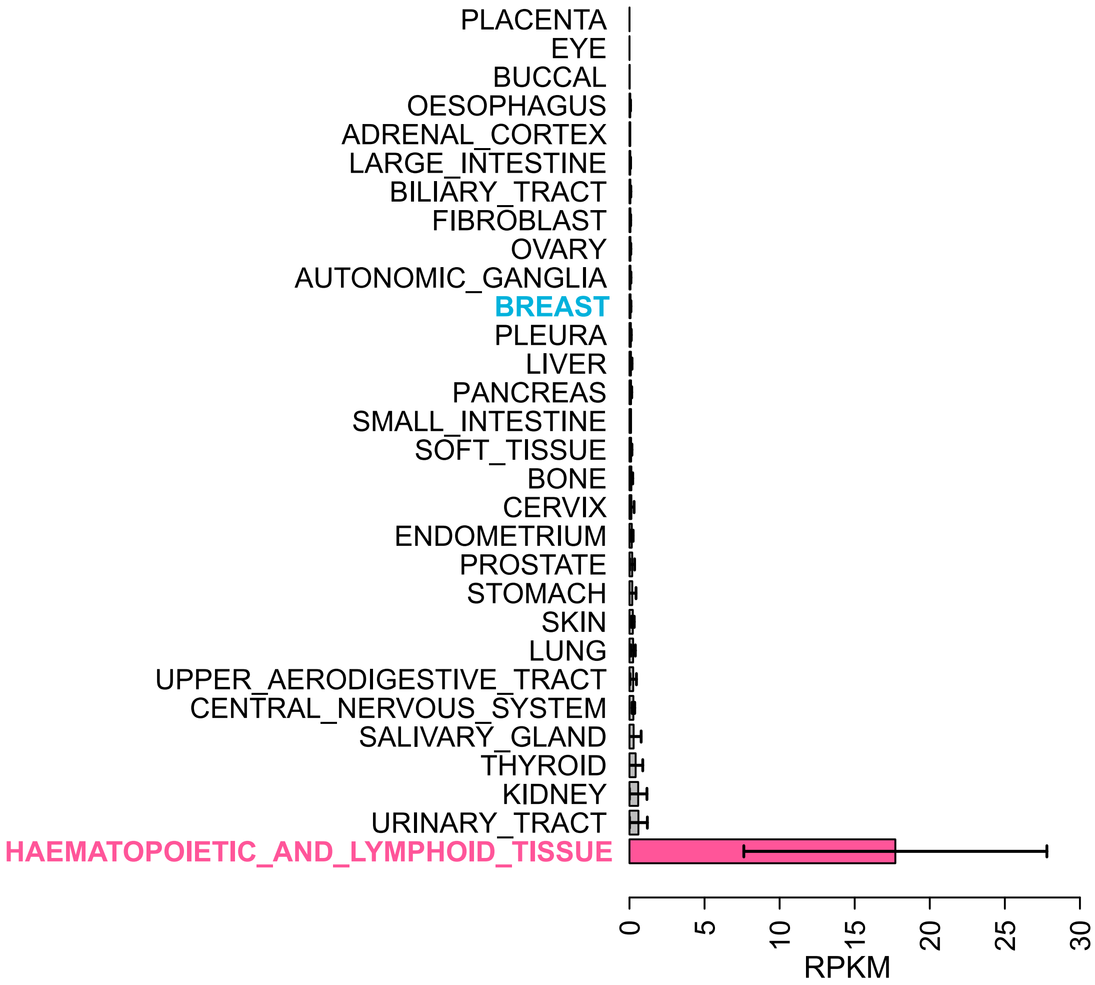

Supplementary Figure 3. *CTSW* expression across different cell lines using data from CCLE. Cell lines are grouped by their tissue type; tissues are ranked based on the mean *CTSW* expression of cell lines within each group. An error bar is also shown for each group indicating standard deviation.

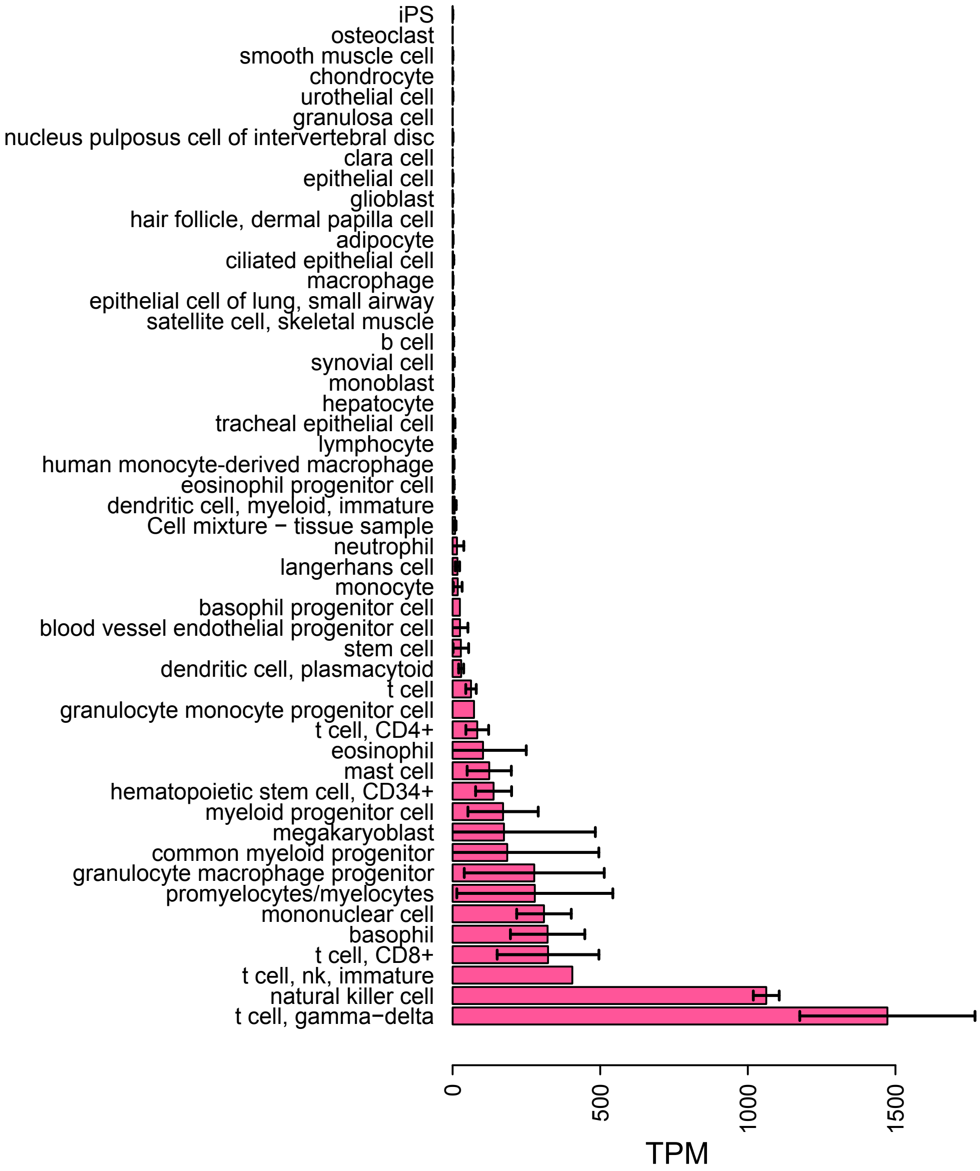

Supplementary Figure 4. The *CTSW* promoter transcription activity measured by FANTOM5 for different cell types. Top 50 cell types are shown, ranked by mean *CTSW* expression within each cell type. The cell types listed are obtained from an annotation file provided by the FANTOM5 consortium. An error bar is also shown for each cell type indicating standard deviation. TPM, tags per million.

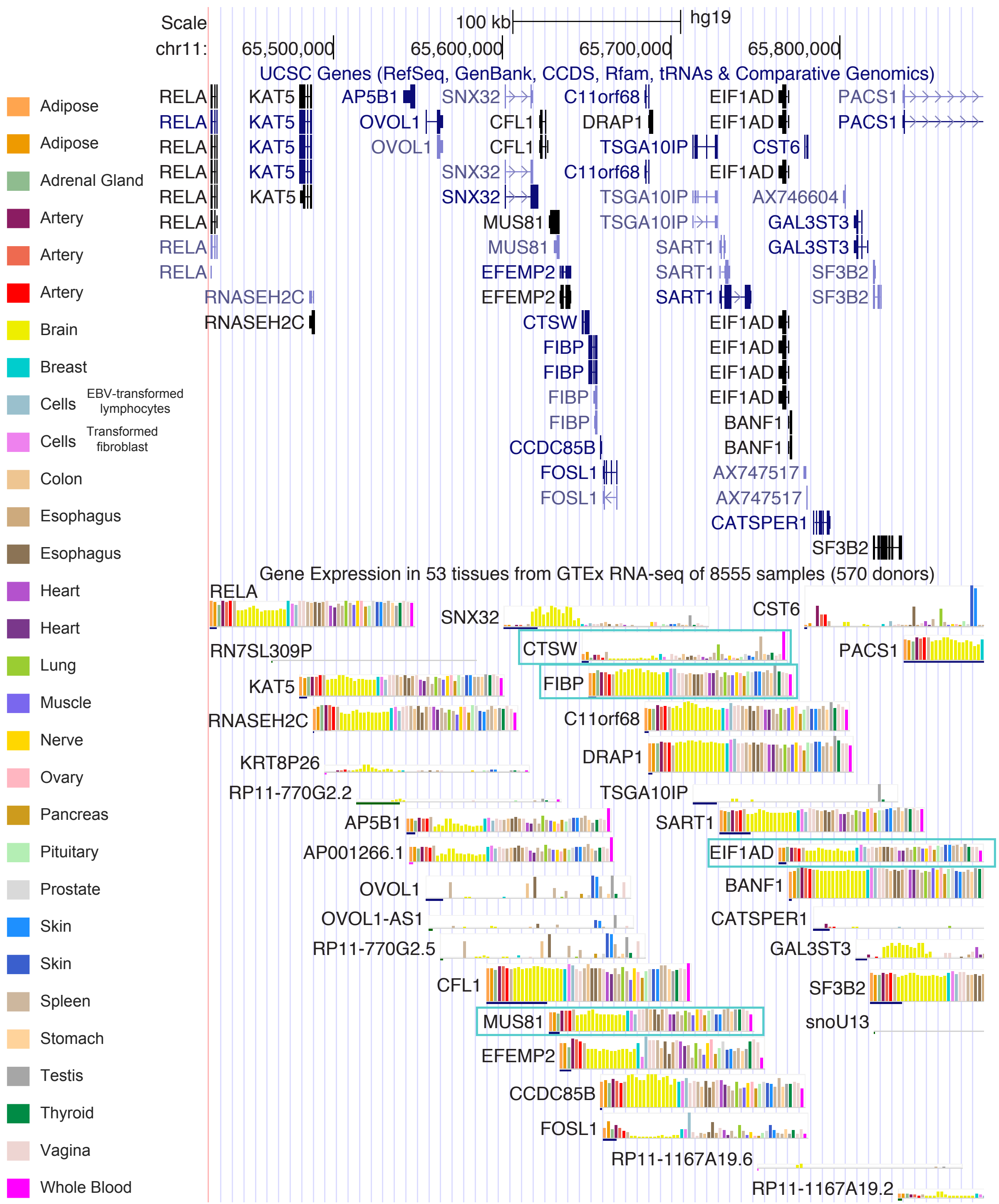

Supplementary Figure 5. Tissue-wise expression of *CTSW* and nearby genes. The four eQTL genes shown in Figure 1B (*MUS81, CTSW, FIBP, EIF1AD*) are boxed. The tissue specificity of *CTSW* expression is clearly visible.

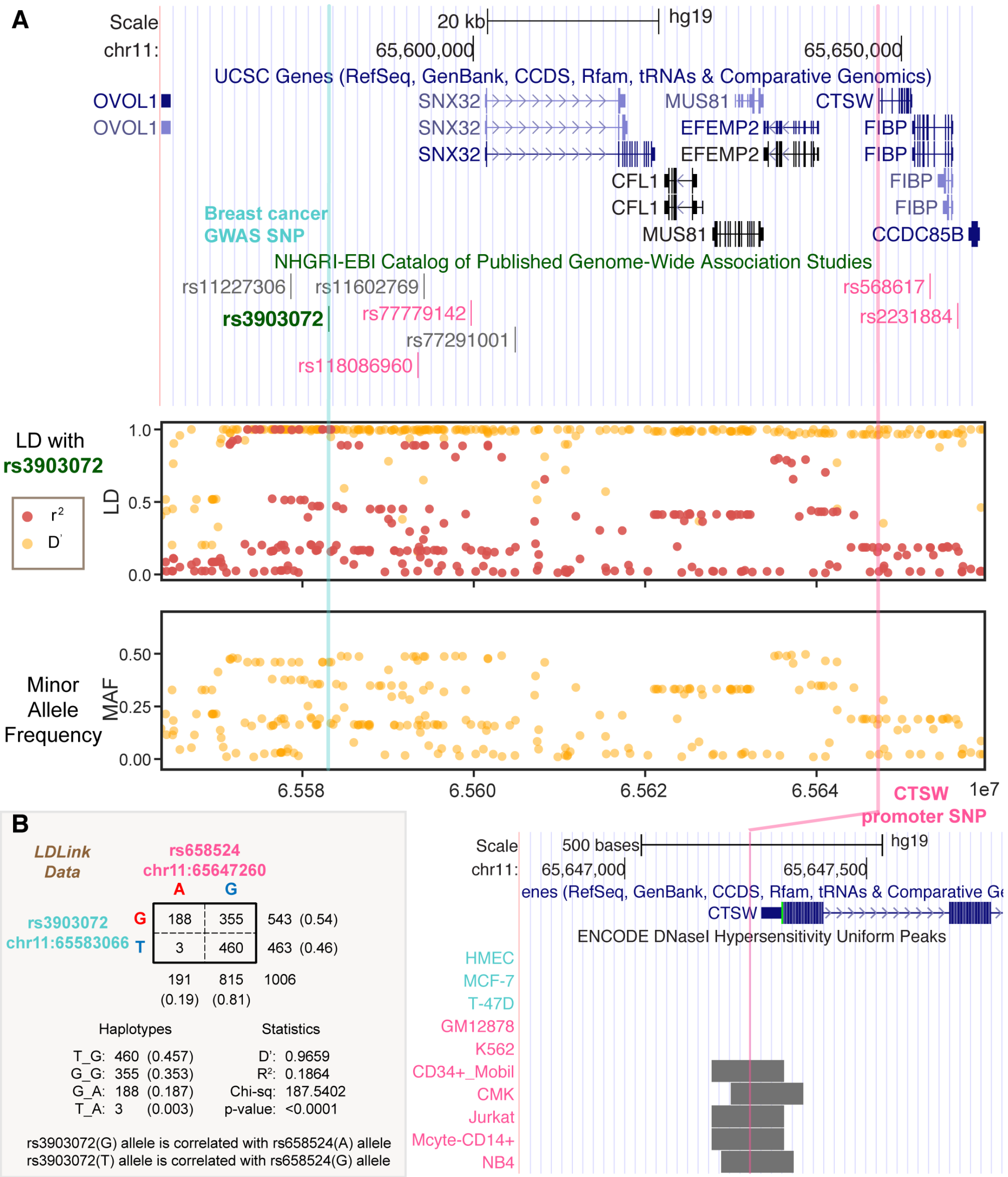

Supplementary Figure 6. The putative regulatory SNP in *CTSW* promoter in weak linkage with the GWAS SNP. (**A**) The *CTSW* promoter SNP is in high D' but low r^2 with the breast cancer GWAS SNP rs3903072, and it is a rarer SNP compared to rs3903072. Enlarging the *CTSW* promoter region shows that the promoter SNP is located near the center of the *CTSW* DHS in several blood cell lines. This is a zoomed-in region of **Figure 1A** where the immuno-inflammatory GWAS variants are marked magenta, with one more Rosacea SNP rs77779142 (**Supplementary Table 1**). (**B**) The linkage structure between the *CTSW* promoter SNP and the GWAS SNP. Most haplotypes carrying the rs658524-A allele have the rs3903072-G (risk) allele (data presentation from LDLink: https://ldlink.nci.nih.gov, computed based on 1000 Genomes phase 3 EUR population).

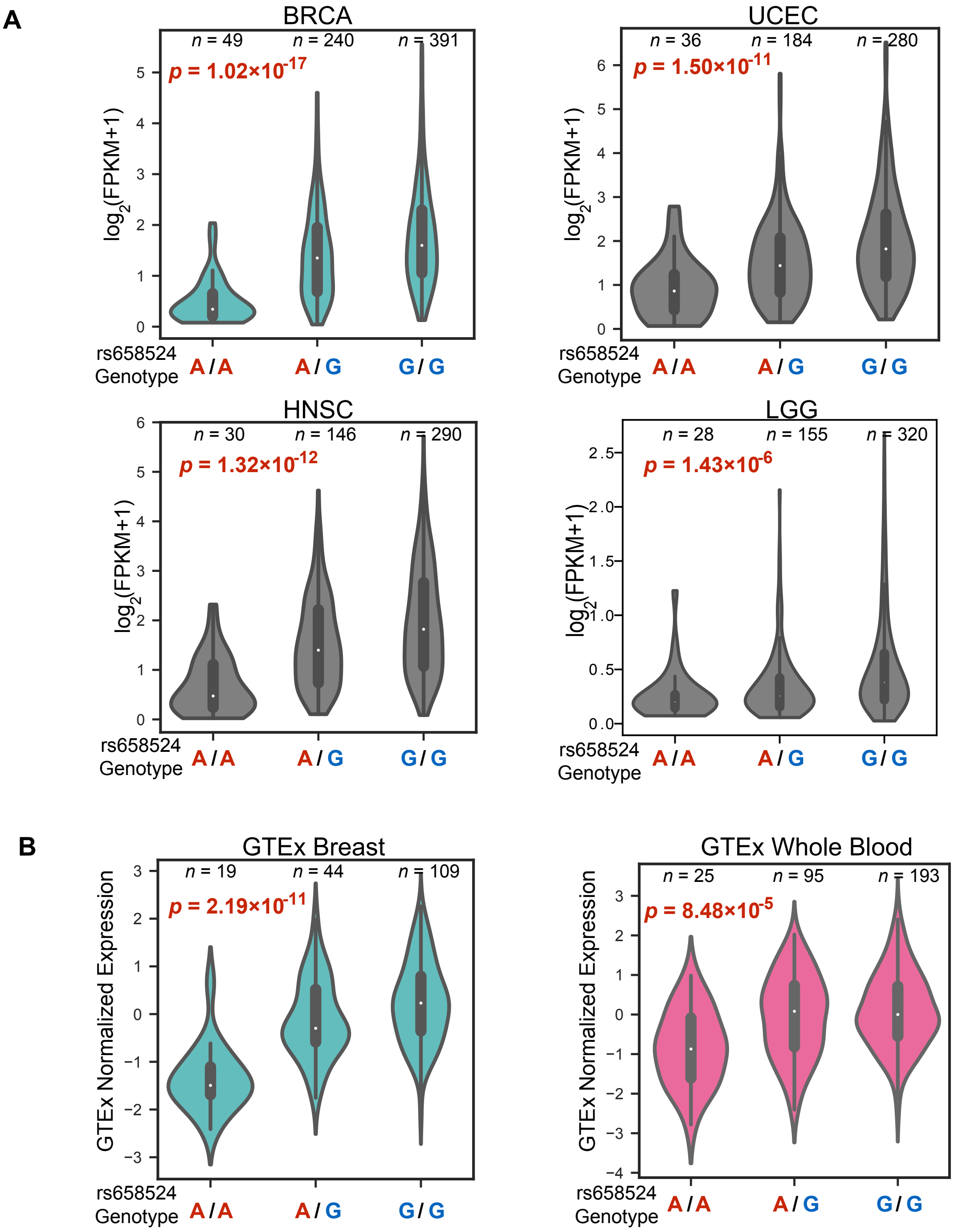

Supplementary Figure 7. Violin plots for the eQTL analysis in different datasets, similar to **Supplementary Figure 2** but using the genotypes at the *CTSW* promoter SNP. A linear model was constructed between the *CTSW* expression level and the genotype status at the promoter SNP rs658524; the *p*-values are for the linear coefficient of genotype, and gene copy number is not included in the model for this figure. Note that rs658524-A is marked as being the risk allele (red), as inferred through its haplotype structure with rs3903072. (**A**) eQTL analysis in cancers from TCGA, using ER+ breast cancer subtype in BRCA, endometrial cancer (UCEC), head and neck cancer (HNSC), and low grade glioma (LGG). (**B**) eQTL analysis in normal tissues from GTEx, using mammary tissue and whole blood tissue.

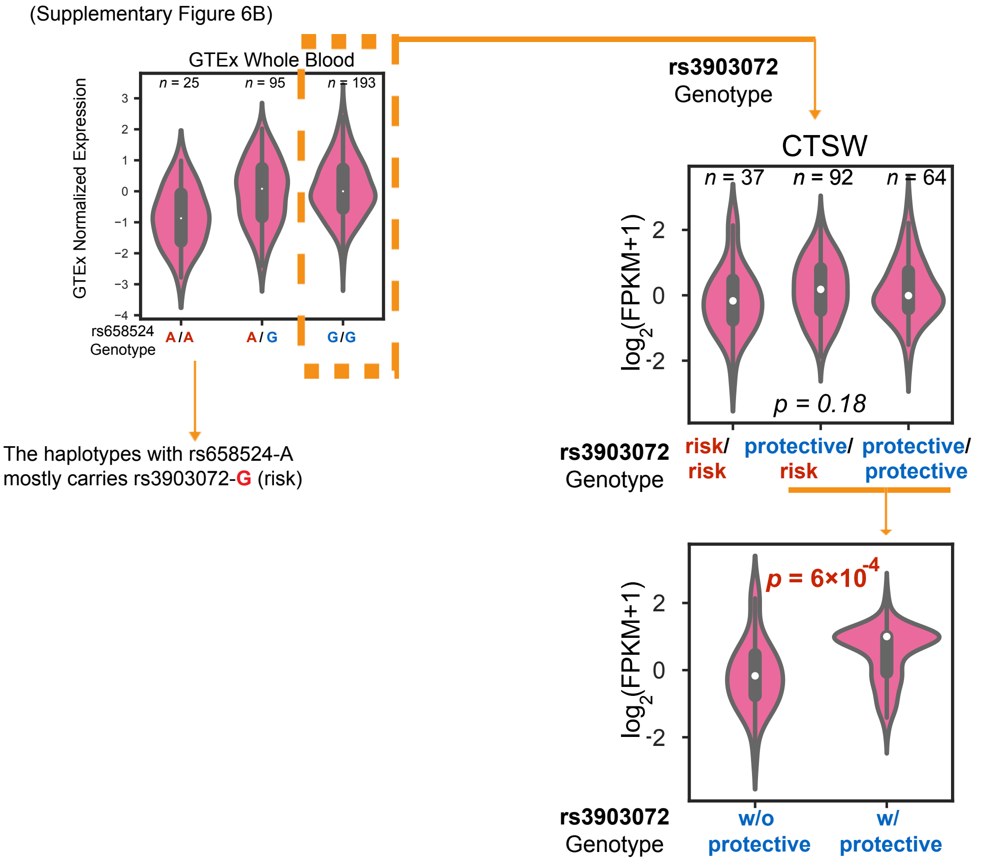

Supplementary Figure 8. eQTL analysis conditioned on the protective homozygous genotype at the *CTSW* promoter SNP. We selected the samples carrying G/G at the promoter SNP, and studied whether there exists a residual effect from the distal GWAS genotype on the *CTSW* expression level. A recessive effect of the GWAS SNP is observed in the bottom plot. The *p*-value in the top right plot is for the genotype coefficient from the eQTL linear model, and the *p*-value in the bottom plot is computed using two-sided Welch's t test.

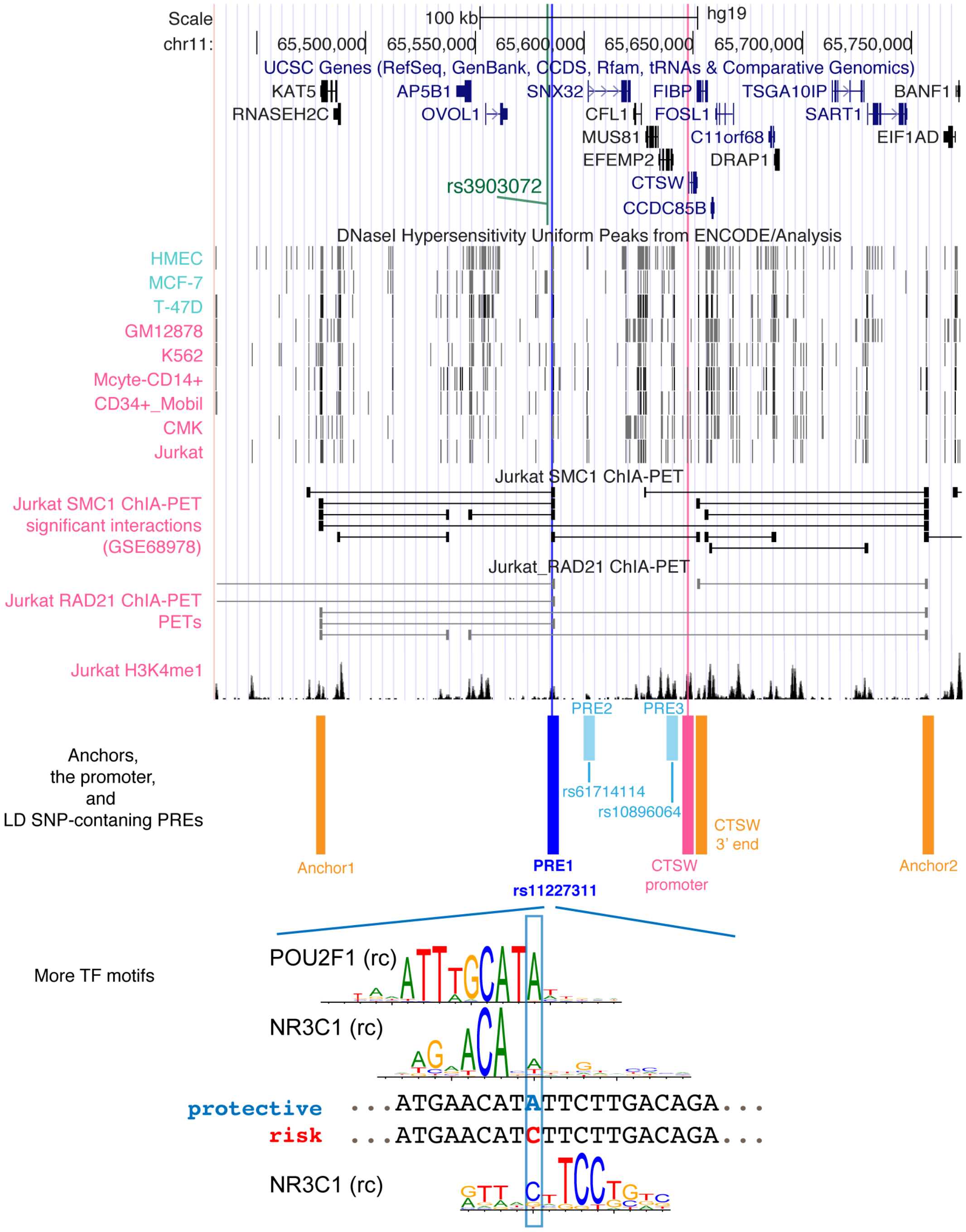

Supplementary Figure 9. ChIA-PET data and anchoring elements around the GWAS region. The same region as in **Figure 1A** is shown, presenting DHS tracks, ChIA-PET data in the Jurkat cell line, putative regulatory elements, and anchoring elements that may mediate multi-way chromatin interactions. The putative regulatory elements (PRE), PRE1, PRE2, and PRE3, all contain GWAS-linked SNPs overlapping a blood cell DHS (**Supplementary Table 5**), but PRE1 is highlighted, because of its interaction with the 3' end of *CTSW*. Several possible TF motifs affected by the PRE1 SNP, besides those displayed in **Figure 3B**, are shown here.

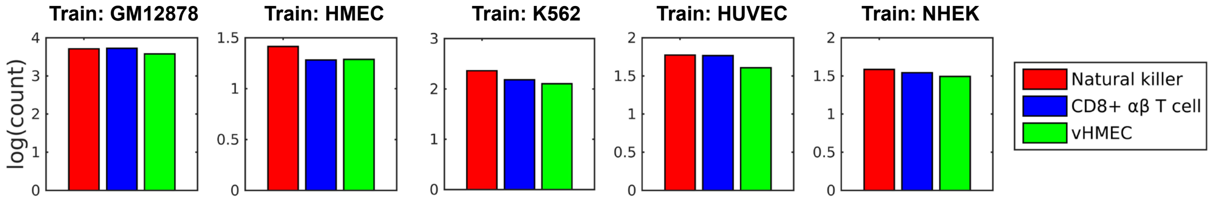

Supplementary Figure 10. Predicted log contact counts for the pair between rs3903072-PRE1 and *CTSW* promoter in three cell lines: Natural Killer cells, CD8+ αβ T cells and vHMEC.

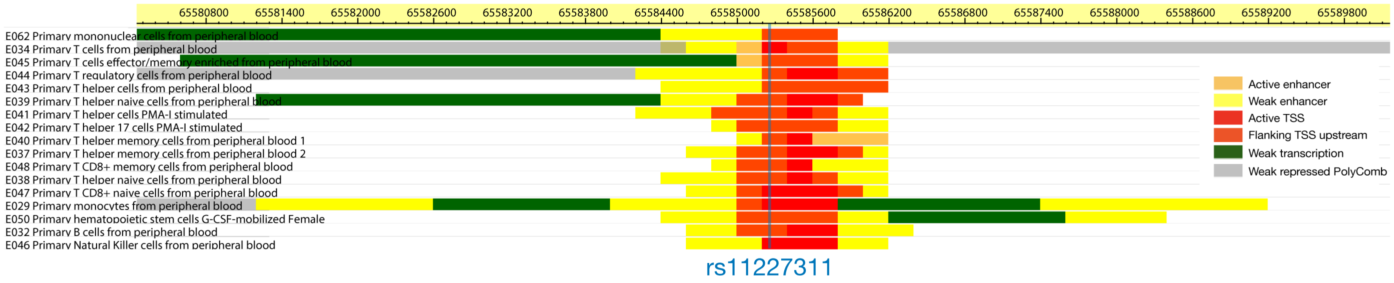

Supplementary Figure 11. Epigenetic state of primary blood cells at PRE1. Data from 18-state ChromHMM in Roadmap Epigenetics from REMC.

References

Barretina, J., Caponigro, G., Stransky, N., Venkatesan, K., Margolin, A.A., Kim, S., et al. (2012). The Cancer Cell Line Encyclopedia enables predictive modelling of anticancer drug sensitivity. *Nature* 483**,** 603. doi: 10.1038/nature11003.

Bernstein, B.E., Stamatoyannopoulos, J.A., Costello, J.F., Ren, B., Milosavljevic, A., Meissner, A., et al. (2010). The NIH Roadmap Epigenomics Mapping Consortium. *Nature biotechnology* 28(10)**,** 1045-1048. doi: 10.1038/nbt1010-1045.

Carithers, L.J., Ardlie, K., Barcus, M., Branton, P.A., Britton, A., Buia, S.A., et al. (2015). A Novel Approach to High-Quality Postmortem Tissue Procurement: The GTEx Project. *Biopreservation and Biobanking* 13(5)**,** 311-319. doi: 10.1089/bio.2015.0032.

Das, S., Forer, L., Schönherr, S., Sidore, C., Locke, A.E., Kwong, A., et al. (2016). Next-generation genotype imputation service and methods. *Nature genetics* 48(10)**,** 1284-1287. doi: 10.1038/ng.3656.

GTEx Consortium (2013). The Genotype-Tissue Expression (GTEx) project. *Nature genetics* 45(6)**,** 580-585. doi: 10.1038/ng.2653.

Kassambara, A., and Kosinski, M. (2017). Survminer: Drawing Survival Curves using 'ggplot2'.

Kent, W.J., Sugnet, C.W., Furey, T.S., Roskin, K.M., Pringle, T.H., Zahler, A.M., et al. (2002). The human genome browser at UCSC. *Genome research* 12(6)**,** 996-1006. doi: 10.1101/gr.229102.

Loh, P.-R., Danecek, P., Palamara, P.F., Fuchsberger, C., A Reshef, Y., K Finucane, H., et al. (2016a). Reference-based phasing using the Haplotype Reference Consortium panel. *Nature genetics* 48(11)**,** 1443-1448. doi: 10.1038/ng.3679.

Loh, P.-R., Palamara, P.F., and Price, A.L. (2016b). Fast and accurate long-range phasing in a UK Biobank cohort. *Nature Genetics* 48**,** 811. doi: 10.1038/ng.3571.

MacArthur, J., Bowler, E., Cerezo, M., Gil, L., Hall, P., Hastings, E., et al. (2017). The new NHGRI-EBI Catalog of published genome-wide association studies (GWAS Catalog). *Nucleic Acids Research* 45(D1)**,** D896-D901. doi: 10.1093/nar/gkw1133.

Malone, J., Holloway, E., Adamusiak, T., Kapushesky, M., Zheng, J., Kolesnikov, N., et al. (2010). Modeling sample variables with an Experimental Factor Ontology. *Bioinformatics* 26(8)**,** 1112-1118. doi: 10.1093/bioinformatics/btq099.

McLean, C.Y., Bristor, D., Hiller, M., Clarke, S.L., Schaar, B.T., Lowe, C.B., et al. (2010). GREAT improves functional interpretation of cis-regulatory regions. *Nature Biotechnology* 28(5)**,** 495-U155. doi: 10.1038/nbt.1630.

Mi, H., Huang, X., Muruganujan, A., Tang, H., Mills, C., Kang, D., et al. (2017). PANTHER version 11: expanded annotation data from Gene Ontology and Reactome pathways, and data analysis tool enhancements. *Nucleic Acids Research* 45(D1)**,** D183-D189. doi: 10.1093/nar/gkw1138.

Michailidou, K., Hall, P., Gonzalez-Neira, A., Ghoussaini, M., Dennis, J., Milne, R.L., et al. (2013). Large-scale genotyping identifies 41 new loci associated with breast cancer risk. *Nature genetics* 45(4)**,** 353-361e3612. doi: 10.1038/ng.2563.

Michailidou, K., Lindström, S., Dennis, J., Beesley, J., Hui, S., Kar, S., et al. (2017). Association analysis identifies 65 new breast cancer risk loci. *Nature* 551**,** 92. doi: 10.1038/nature24284.

Rao, S.S.P., Huntley, M.H., Durand, N.C., Stamenova, E.K., Bochkov, I.D., Robinson, J.T., et al. (2014). A 3D map of the human genome at kilobase resolution reveals principles of chromatin looping. *Cell* 159(7)**,** 1665-1680. doi: 10.1016/j.cell.2014.11.021.

Schreiber, J., Durham, T.J., Bilmes, J., and Noble, W.S. (2018). Multi-scale deep tensor factorization learns a latent representation of the human epigenome. *bioRxiv***,** 364976. doi: 10.1101/364976.

Sherwood, R.I., Hashimoto, T., O'Donnell, C.W., Lewis, S., Barkal, A.A., van Hoff, J.P., et al. (2014). Discovery of directional and nondirectional pioneer transcription factors by modeling DNase profile magnitude and shape. *Nature Biotechnology* 32**,** 171. doi: 10.1038/nbt.2798.

The Encode Project Consortium, Dunham, I., Kundaje, A., Aldred, S.F., Collins, P.J., Davis, C.A., et al. (2012). An integrated encyclopedia of DNA elements in the human genome. *Nature* 489**,** 57. doi: 10.1038/nature11247.

The Fantom Consortium and the Riken PMI and CLST (DGT), Forrest, A.R.R., Kawaji, H., Rehli, M., Kenneth Baillie, J., de Hoon, M.J.L., et al. (2014). A promoter-level mammalian expression atlas. *Nature* 507**,** 462. doi: 10.1038/nature13182.

Therneau, T. (2015). A Package for Survival Analysis in S. version 2.38.

Uhlen, M., Zhang, C., Lee, S., Sjöstedt, E., Fagerberg, L., Bidkhori, G., et al. (2017). A pathology atlas of the human cancer transcriptome. *Science* 357(6352)**,** eaan2507.

Whalen, S., Truty, R.M., and Pollard, K.S. (2016). Enhancer–promoter interactions are encoded by complex genomic signatures on looping chromatin. *Nature Genetics* 48**,** 488. doi: 10.1038/ng.3539.

Wu, C., Jin, X., Tsueng, G., Afrasiabi, C., and Su, A.I. (2016). BioGPS: building your own mash-up of gene annotations and expression profiles. *Nucleic acids research* 44(D1)**,** D313-D316. doi: 10.1093/nar/gkv1104.

Zhang, S., Chasman, D., Knaack, S., and Roy, S. (2018a). In silico prediction of high-resolution Hi-C interaction matrices. *bioRxiv***,** 406322. doi: 10.1101/406322.

Zhang, Y., Manjunath, M., Zhang, S., Chasman, D., Roy, S., and Song, J.S. (2018b). Integrative Genomic Analysis Predicts Causative Cis-Regulatory Mechanisms of the Breast Cancer-Associated Genetic Variant rs4415084. *Cancer Research* 78(7)**,** 1579-1591. doi: 10.1158/0008-5472.CAN-17-3486.
